# Transglutaminase 2 Induces Deficits in Social Behavior in Mice

**DOI:** 10.1155/2018/2019091

**Published:** 2018-12-13

**Authors:** Amanda Crider, Talisha Davis, Anthony O. Ahmed, Lin Mei, Anilkumar Pillai

**Affiliations:** ^1^Department of Psychiatry and Health Behavior, Augusta University, Augusta, GA 30912, USA; ^2^Department of Psychiatry, Weill Cornell Medical College, White Plains, NY, USA; ^3^Department of Neurosciences, School of Medicine, Case Western Reserve University, Cleveland, OH 44106, USA

## Abstract

Impairments in social behavior are highly implicated in many neuropsychiatric disorders. Recent studies indicate a role for endoplasmic reticulum (ER) stress in altering social behavior, but the underlying mechanism is not known. In the present study, we examined the role of transglutaminase 2 (TG2), a calcium-dependent enzyme known to be induced following ER stress, in social behavior in mice. ER stress induced by tunicamycin administration increased TG2 protein levels in the mouse prefrontal cortex (PFC). PFC-specific inhibition of TG2 attenuated ER stress-induced deficits in social behavior. Conversely, overexpression of TG2 in the PFC resulted in social behavior impairments in mice. In addition, systemic administration of cysteamine, a TG2 inhibitor, attenuated social behavior deficits. Our preliminary findings using postmortem human brain samples found increases in TG2 mRNA and protein levels in the middle frontal gyrus of subjects with autism spectrum disorder. These findings in mice and human postmortem brain samples identify changes in TG2 activity in the possible dysregulation of social behavior.

## 1. Introduction

Deficits in social behavior are core symptoms of many psychiatric and neurodevelopmental disorders including major depression, autism spectrum disorder (ASD), and schizophrenia [1]. Although the underlying neurobiological mechanisms are not well understood, various genetic as well as epigenetic factors have been shown to play critical roles in social behaviors [2]. Recently, accumulating data have implicated disruption of endoplasmic reticulum (ER) homeostasis, or ER stress, in the pathophysiology of the above disorders [3–7].

The ER is an intracellular organelle responsible for folding, maturation, quality control, and traffic of secretory or transmembrane proteins [8]. A number of conditions including nutrient deprivation, hypoxia, change in calcium homeostasis, viral infections, environmental toxins, inflammatory cytokines, and genetic mutations can provoke an accumulation of improperly folded proteins in this compartment, thereby causing ER stress and triggering the unfolded protein response (UPR) [9, 10]. Three ER-resident proteins have been identified as sensors of ER stress: IRE1 (inositol-requiring protein 1), PERK (PKR- (double-stranded RNA-dependent protein kinase) like ER kinase), and ATF6 (activating transcription factor) [11]. IRE1 is a type 1 transmembrane serine/threonine receptor protein kinase which functions as a sensor for misfolded/unfolded proteins in the ER lumen. Activated IRE1 induces the splicing of *XBP1* (X-box-binding protein 1) mRNA by cleaving off its intron [12]. The UPR generally acts as a prosurvival mechanism, mediated by translation arrest and the induction of a number of transcription factors and chaperone proteins that function to restore ER homeostasis. However, when ER stress is prolonged or the degree of ER stress is too severe, UPR signaling can initiate programmed cell death by activating stress-induced proapoptotic mechanisms [13, 14].

In a recent study, we found that treatment with tunicamycin, an ER stress inducer, enhanced the phosphorylation level of IRE1 and increased X-box-binding protein 1 (XBP1) mRNA splicing activity in the mouse PFC, whereas inhibition of IRE1/XBP1 pathway in the PFC attenuated social behavioral deficits caused by tunicamycin treatment [15]. However, the cellular mechanism involved in ER stress-induced social behavior deficits is not clear. It is known that the ER is an important source of Ca^2+^ necessary for regulating a variety of cellular functions both in the ER lumen and in the cytosol [16]. Moreover, disruption of this Ca^2+^ homeostasis has adverse effects on cellular functions. Although the activation of cell death pathways usually requires prolonged ER stress and disrupted Ca^2+^ gradients, many cytosolic Ca^2+^-dependent enzymes are activated in response to transient ER stress and increased cytosolic Ca^2+^ concentrations [17]. In this regard, tissue transglutaminase 2 (TG2) is a ubiquitously expressed Ca^2+^-dependent enzyme that resides in the cytosol [18]. Increase in Ca^2+^ concentration in the cytosol such as when Ca^2+^ is released from the ER during ER stress has been shown to activate TG2 [19]. TG2 is involved in many critical physiological functions including cell adhesion, growth, migration, differentiation, programmed cell death, and ECM assembly [20]. In the CNS, TG2 has been shown to play an important role in neural development and functioning [21]. Our recent study showed that chronic stress induces TG2 levels in the PFC of mice, and TG2 levels are higher in the PFC of depressed suicide subjects [22]. Moreover, pharmacological inhibition of TG2 using cysteamine attenuated anxiety- and depression-like behavioral abnormalities induced by chronic corticosterone treatment in mice [23]. Cysteamine is an FDA-approved drug currently prescribed for cystinosis and has neuroprotective as well as antioxidant properties [24]. Cysteamine has been shown to increase brain as well as serum BDNF levels in rodents [25, 26]. Further, our recent study has shown that cysteamine increases TrkB signaling in the mouse frontal cortex [23].

In the present study, we investigated the role of TG2 in ER stress-induced deficits in social behavior in mice. Since our recent study [7] found a significant increase in ER stress-related proteins in the middle frontal gyrus, a PFC region highly implicated in ASD [7, 27–29] of ASD subjects, we examined whether TG2 levels are altered in the above brain region of ASD subjects.

## 2. Materials and Methods

### 2.1. Ethics Statement

The Augusta University Institutional Review Board has deemed this study exempt from full review due to the use of deidentified human postmortem brain samples, with no possibility to track back the identity of the donors. Human postmortem samples are from the NICHD Brain and Tissue Bank for Developmental Disorders at the University of Maryland with ethical permission granted by the institutional review boards of the University of Maryland. Animal studies were carried out in compliance with the US National Institute of Health guidelines and approved by Augusta University animal welfare guidelines.

#### 2.1.1. Animals

Adult (8–10 weeks old) C57BL/6J male mice were purchased from Charles River Laboratories (Wilmington, MA, USA). Mice were housed in groups of 4 mice in standard polypropylene cages in 12 h light-dark cycle. All behavior experiments were performed at 8–10 weeks of age. The same animals that were used for behavioral analysis were used for molecular studies.

#### 2.1.2. Drug Treatment

Mice were injected intraperitoneally (i.p.) with 1 mg/kg tunicamycin (catalog #T7765; Sigma, St. Louis, Missouri) dissolved in DMSO (vehicle control). Tunicamycin is known to cross blood-brain barrier [30], and tunicamycin administration (1 mg/kg; i.p.) has been shown to induce increases in ER stress markers in the mouse brain [31, 32]. Our previous study has shown that tunicamycin treatment for 12 h induces deficits in social interaction in mice [15]. Cysteamine (Sigma) was dissolved in water. Cysteamine (150 mg/kg; i.p.) was given 30 min before tunicamycin treatment. The cysteamine dose was selected based on earlier studies where the above concentration was found to be nontoxic, but showed neuroprotective effects [23, 25].

#### 2.1.3. Stereotaxic Injection of Lentivirus

pLenti-GIII-CMV-mTGM2-GFP-2A-Puro, pLenti-CMV-GFP-2A-Puro-Blank vector, lentiviral vector expressing TG2 siRNA, and scrambled siRNA GFP were purchased from Applied Biological Materials Inc. [22]. A total volume of 1.0 *μ*l (1 × 10^9^ infectious particles per milliliter) of lentivirus was administered into the mouse PFC (anterior-posterior (AP) = +1.8 mm; mediolateral (ML) = 0 mm; dorsoventral (DV) = −2.5 mm) by stereotaxic microinjection at a rate of 0.2 *μ*l/min at each site (Stoelting Co.) [22]. Tunicamycin administration was performed two weeks following lentiviral injection. Behavior studies were performed 12 h post-tunicamycin treatment.

#### 2.1.4. Behavior Experiments

Behavioral testing was performed in a room with constant background sound and ambient lighting approximately 25–30 lux (lumen/m^2^) unless noted. Temperature and pressure in behavioral rooms are monitored and kept constant. Animals are transferred in their home cages to behavioral rooms at least 1 hour before testing and allowed to habituate to the testing room. All behavioral experiments were scored blind to treatment.

#### 2.1.5. Three-Chamber Test

This test was performed to measure sociability and social deficits. The test mouse was placed in a box with 3 chambers. Each chamber is 19 cm × 45 cm × 22 cm, and the dividing walls are made from clear Plexiglas®, with openings on each wall for free access to the other two chambers. Two identical wire containers that were large enough to house a single mouse were placed vertically inside the apparatus with one in each side chamber and weighted down. The test mouse was habituated to the apparatus for 5 minutes while freely exploring. After the habituation period, the stranger mouse was placed in one of the wire cup-like containers (diameter: 9 cm) while the test mouse was still allowed to freely move outside of the container. The wire containers allow air exchange between the interior and exterior, but the holes are small enough to prevent direct physical contact between the stranger mouse and test mouse. The free test mouse was allowed to interact through the wire container with the stranger mouse for 5 minutes. During this time, time spent in chambers (stranger mouse, empty cage, and center) was video recorded. The stranger mouse chamber is defined as the chamber containing the wire container with the stranger mouse inside. The empty cage chamber is the chamber containing an empty wire container. The stranger mouse was a mouse of similar age, same sex, and similar weight as the test mouse.

#### 2.1.6. Reciprocal Social Interaction Test

This test was performed to measure social approach behavior. The test mouse was placed in a neutral box (57 cm × 45 cm × 22 cm) made from clear Plexiglas® and allowed to habituate for 5 minutes. After habituation, a stranger mouse was placed in the box and the test mouse was allowed to freely interact with the stranger mouse. Interaction is defined as close physical contact, nose-to-nose sniffing, anogenital sniffing, and grooming. Time spent interacting (initiated by the test mouse) was video recorded. The stranger mouse was a mouse of similar age, same sex, and similar weight as the test mouse.

#### 2.1.7. Western Blotting

Mice were sacrificed by cervical dislocation after being anesthetized using isoflurane. To dissect the PFC area, cuts were made on the medial side of both hemispheres at approximately bregma 2 and interaural 3. This region includes all or parts of the frontal association cortex, orbital cortex, prelimbic cortex, motor cortex, insular cortex, and cingulate cortex. To dissect the hippocampus, the diencephalon and brain stem were removed to access the medial aspect of the telencephalon. The hippocampal formation was then “rolled out” from the rest of the telencephalon. This region includes the entire dorsal-to-ventral extent of the hippocampal formation with the dentate gyrus, CA3, CA2, CA1, and subiculum. PFC and hippocampus tissues from mice or postmortem human middle frontal gyrus samples were homogenized in a tissue lysis buffer containing 50 mM Tris-HCl (pH 7.5), 150 mM NaCl, 1.0% sodium deoxycholate, 0.1% sodium dodecyl sulfate (SDS), 2 mM EDTA, 6 *μ*M PMSF, and 1.0% Triton X-100 supplemented with protease inhibitor cocktail (Sigma, St. Louis, Missouri). The homogenate was centrifuged at 13,000 rpm for 10 min at 4°C, and the supernatant was used for protein estimation by the bicinchoninic acid method (BCA Protein Assay Kit, Sigma, St. Louis, Missouri). Samples (30 *μ*g) were subjected to SDS-PAGE and transferred onto a nitrocellulose membrane. The membrane was blocked for 1 hour in PBS with Tween 20 and 5%–10% nonfat milk followed by overnight incubation with a primary antibody. Blots were incubated in the appropriate primary antibody specific for TG2 (Cell Signaling; 1 : 1000), tubulin (Cell Signaling, Boston, MA; 1 : 10,000), actin (Sigma; 1 : 5000), or GAPDH (Cell Signaling; 1 : 5000) and developed with the SuperSignal West Pico Chemiluminescent substrate system (Thermo Fisher Scientific, West Columbia, SC). Optical densities of the bands were analyzed using the ImageJ software (NIH). For analysis, protein levels were normalized to housekeeping protein levels and then expressed as a fold change of that in control animals. For figure panels, contrasts have been adjusted linearly for easier viewing of bands.

### 2.2. Human Postmortem Samples

The postmortem sample comprised 13 ASD subjects and 13 controls. Demographic information is included in Table 1. Autism Diagnostic Interview-Revised (ADI-R) scores were available for 9 out of the 13 ASD subjects. There were no significant differences between tissues of ASD and control subjects in the areas of PMI, refrigeration interval, age, RNA integrity, and brain pH (Table 1).

#### 2.2.1. Quantitative Reverse Transcriptase PCR (qRT-PCR)

RNA was purified using a commercially available kit (SV RNA Isolation, Promega, Madison, WI, USA); qRT-PCR was performed on a Mastercycler (Eppendorf, Hamburg, Germany) using a SuperScript III Platinum SYBR Green One-Step qRT-PCR kit (Invitrogen, Carlsbad, CA, USA). TG2 primers used were forward: 5′-tcaactgcaacgatgaccagg-3′ and reverse: 5′-tgttctggtcatgggccg-3′ (Integrated DNA Technologies). Ct values were normalized to the geometric mean of two control genes (*β*-actin and GAPDH).

### 2.3. Data Analysis

All analyses of the data were completed using the IBM SPSS statistical software (version 20). All data are presented as mean ± SEM (error bars). For mouse behavioral studies, we used a two-way ANOVA or one-way ANOVA with a Bonferroni multiple comparison post hoc test unless otherwise specified in the figure legend. In human postmortem data analysis, analysis of covariance (ANCOVA) models served to compare TG2 mRNA and protein levels in the postmortem samples of people with ASD and healthy controls. Differences were examined with affection status (ASD versus controls) entered into the model as between-subject factor and age, postmortem interval (PMI), pH, storage time, and RNA integrity evaluated for inclusion as possible covariates. TG2 mRNA and TG2 protein expressions were included as dependent variable in separate ANCOVA models. It was determined *a priori* that only covariates with at least small (*r* ≥ 0.20) and/or significant associations with the dependent variable would be included in the ANCOVA as covariate. Exact probability (*p*) values of less than 5% were flagged as statistically significant. Cohen's *d* was computed as a measure of effect size differences between ASD and control subjects.

## 3. Results

### 3.1. TG2 Inhibition in the PFC Attenuates ER Stress-Induced Social Interaction Deficits in Mice

**Figure 1:**
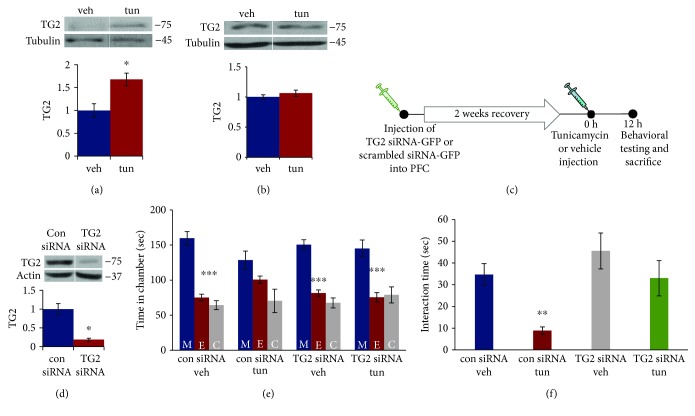
TG2 in the PFC mediates ER stress-induced deficits in social behavior in male mice. Tunicamycin treatment induced increase of TG2 protein levels in the mouse PFC. TG2 protein levels were determined in the mouse (a) prefrontal cortex (PFC) and (b) hippocampus 12 h after tunicamycin injection. Top: representative blot. Bottom: quantification of TG2 normalized to tubulin. Protein levels were measured by western blot analysis. ^∗^*p* < 0.05; Student's *t*-test. *N* = 3 (vehicle) and 5 (tunicamycin). (c) Schematic representation of stereotaxic injection of control or TG2 siRNA lentiviral particles into the mouse PFC followed by tunicamycin treatment for 12 h. (d) Decrease in TG2 expression in the PFC of mice injected with TG2 siRNA particles. Top: representative blot. Bottom: quantification of TG2 normalized to actin. Protein levels were measured by western blot analysis. ^∗^*p* < 0.05; Student's *t*-test. *N* = 4 (control siRNA) and 3 (TG2 siRNA). (e–f) TG2 siRNA administration attenuated tunicamycin-induced deficits in social behavior. (e) The three-chamber social interaction test. ^∗∗∗^*p* < 0.001 (E vs. M); two-way ANOVA. (f) Reciprocal social interaction test. ^∗∗^*p* < 0.01 vs. con siRNA-vehicle group; two-way ANOVA. (e–f) *N* = 7 (con siRNA veh), 7 (con siRNA tun), 7 (TG2 siRNA veh), and 6 (TG2 siRNA tun). Data are expressed as mean ± SEM. M: chamber housing stranger mouse; E: chamber housing an empty cage; C: center.

To determine whether ER stress induces TG2 levels in the brain, we examined TG2 protein levels in the PFC and hippocampus (two key brain regions involved in social behavior) of mice exposed to tunicamycin treatment. We found a significant increase in TG2 protein levels in the PFC of mice treated with tunicamycin (Figure 1(a); *p* < 0.05). However, no significant change in TG2 protein levels was found in the hippocampus (Figure 1(b)) following tunicamycin treatment. To examine the direct role of TG2 in ER stress-induced social interaction deficits, we silenced TG2 expression in the mouse PFC using lentiviral vectors expressing TG2 siRNA (Figure 1(c)). A significant reduction in TG2 protein levels was found in the mouse PFC following TG2 siRNA administration (*p* < 0.05; Figure 1(d)). In a three-chamber test, two-way ANOVA was performed to determine the time spent in each side chamber (stranger mouse, empty cage, and center). We found significant main effect of chamber (*F*(2, 69) = 74.42, *p* < 0.001), but no significant effects of treatment or chamber *×* treatment interaction in a three-chamber test. Mice exposed to vehicle spent more time in the chamber housing stranger mouse than the empty cage chamber, whereas mice exposed to tunicamycin had no preference for either chamber (Figure 1(e)). However, the above tunicamycin-induced deficit in social behavior was not found in TG2 siRNA-injected mice (*p* < 0.05; Figure 1(e)). In the reciprocal interaction test, a two-way ANOVA of interaction time showed significant main effects of siRNA type (*F*(1, 24) = 7.53, *p* < 0.05) and treatment (*F*(1, 24) = 9.08, *p* < 0.01). Control siRNA-treated mice exposed to tunicamycin showed decreased interaction with a stranger mouse when compared with those from the TG2 siRNA-injected group (*p* < 0.05; Figure 1(f)). These results indicate the important role of TG2 in the PFC in ER stress-induced changes in social behavior.

### 3.2. TG2 Overexpression in the PFC Induces Social Interaction Deficits in Mice

**Figure 2:**
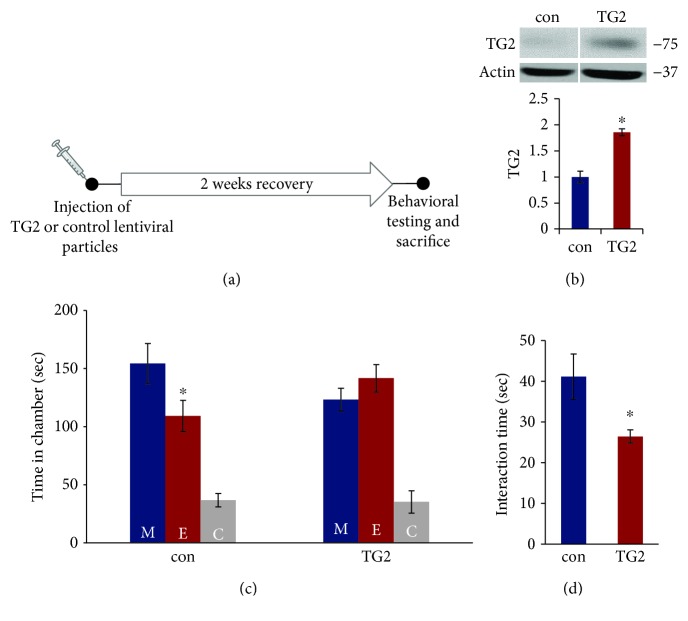
TG2 overexpression in the PFC induces social interaction deficits in mice. (a) Schematic representation of stereotaxic injection of control or TG2 lentiviral particles into the mouse PFC. (b) Increase in TG2 expression in the PFC of mice injected with TG2 lentiviral particles. Top: representative blot. Bottom: quantification of TG2 normalized to actin. Protein levels were measured by western blot analysis. ^∗^*p* < 0.05; Student's *t*-test. *N* = 3 (control) and 4 (TG2). (c–d) TG2 overexpression induced deficits in social behavior. (c) The three-chamber social interaction test. ^∗^*p* < 0.05 (E vs. M); two-way ANOVA. (d) Reciprocal social interaction test. ^∗^*p* < 0.05 vs. con group; one-way ANOVA. *N* = 7 mice per group. Data are expressed as mean ± SEM. M: chamber housing stranger mouse; E: chamber housing an empty cage; C: center.

The role of TG2 in social behavior was further examined by determining social behavior in mice overexpressing TG2 in the PFC. We overexpressed TG2 in the mouse PFC by injecting TG2 lentiviral particles [22] and examined social behavior two weeks later (Figure 2(a)). We found significant increase in TG2 protein levels in the PFC following TG2 overexpression (*p* < 0.05; Figure 2(b)). Two-way ANOVA showed significant effects of time in chamber (*F*(2, 36) = 44.23, *p* < 0.001) and chamber *×* treatment interaction (*F*(2, 36) = 3.75, *p* < 0.05) in the three-chamber test (Figure 2(c)). No significant effect of treatment was found in the two-way ANOVA. Mice injected with control lentiviral particles spent more time in the chamber housing stranger mouse than the empty cage chamber, whereas mice overexpressed with TG2 lentiviral particles had no preference for either chamber (*p* < 0.05; Figure 2(c)). Similarly, TG2-overexpressed mice displayed decreased interaction with a stranger mouse when compared with those from the control group in the reciprocal interaction test (*p* < 0.05; Figure 2(d)). These results further suggest that TG2 in the PFC plays a critical role in social behavior in mice.

### 3.3. TG2 Inhibitor, Cysteamine, Attenuates ER Stress-Induced Social Interaction Deficits in Mice

**Figure 3:**
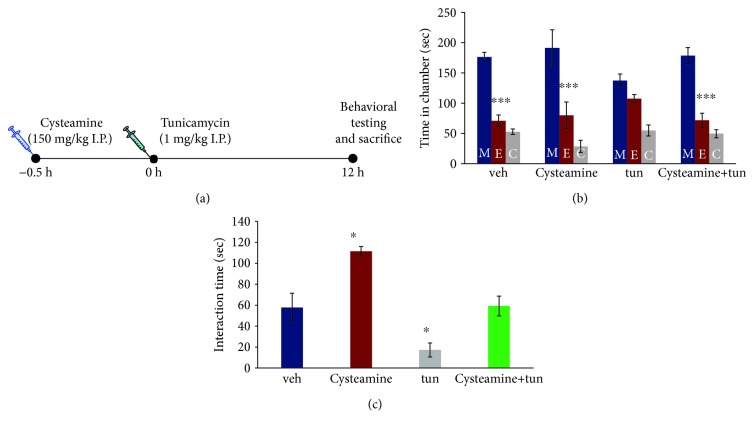
TG2 inhibitor, cysteamine, attenuates ER stress-induced social interaction deficits in mice. (a) Treatment paradigm. Adult male mice were injected intraperitoneally with tunicamycin (1 mg/kg in dimethyl sulfoxide, DMSO), vehicle (DMSO), or cysteamine (cys; 150 mg/kg, 30 minutes before tunicamycin injection) and tunicamycin (1 mg/kg in DMSO). Behavior was performed 12 hours after tunicamycin injection. (b–c) Cysteamine pretreatment attenuated tunicamycin-induced deficits in social behavior. (b) The three-chamber social interaction test. ^∗∗∗^*p* < 0.001 (E vs. M); two-way ANOVA. (c) Reciprocal social interaction test. ^∗^*p* < 0.05 vs. veh group; two-way ANOVA. (b–c) *N* = 6 (vehicle); 6 (tun), 4 (cysteamine), and 6 (cysteamine + tun). Data are expressed as mean ± SEM. M: chamber housing stranger mouse; E: chamber housing an empty cage; C: center.

**Figure 4:**
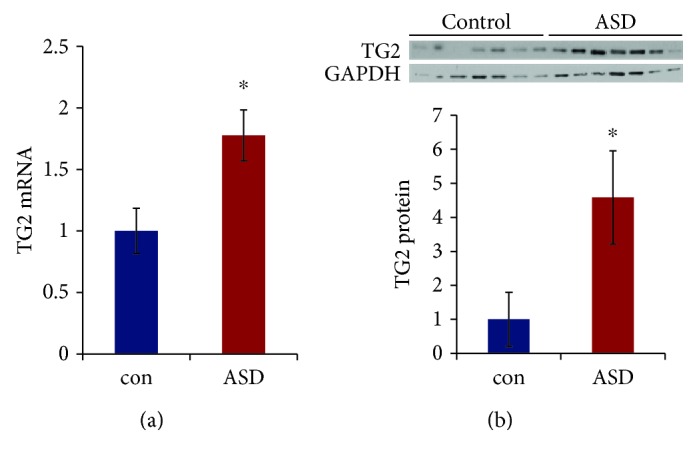
TG2 mRNA and protein levels are higher in the middle frontal gyrus of ASD subjects. (a) Increase in TG2 mRNA in the ASD subjects. mRNA levels were determined by qRT-PCR, and the values were normalized to the geometric mean of two control genes (GAPDH and *β*-actin). ^∗^*p* < 0.05 vs. controls. (b) Increase in TG2 protein levels in the ASD subjects. Top: representative blot. Bottom: quantification of TG2 normalized to GAPDH. Protein levels were measured by western blot analysis. ^∗^*p* < 0.05; Student's *t*-test. *N* = 13.

To determine translational relevance of the above findings on the role of TG2 in social behavior, we examined whether pharmacological inhibition of TG2 using cysteamine could attenuate ER stress-induced social behavior deficits. Adult mice were treated with cysteamine 30 min prior to tunicamycin injection, and behavioral tests were performed 12 h later (Figure 3(a)). Two-way ANOVA showed significant effects of time in chamber (*F*(2, 54) = 120, *p* < 0.001) and chamber × treatment interaction (*F*(6, 54) = 3.6, *p* < 0.01) in the three-chamber test (Figure 4(b)). No significant effect of treatment was found in the two-way ANOVA. Post hoc analysis revealed that cysteamine pretreatment significantly attenuates tunicamycin-induced deficits in social behavior in the three-chamber test (vehicle: *t* = 6.74, *p* < 0.001; cysteamine: *t* = 5.803, *p* < 0.001; tun: *t* = 1.895, *p* > 0.05; and cysteamine + tun: *t* = 6.814, *p* < 0.001) (Figure 3(b)). In the reciprocal interaction test, a two-way ANOVA of interaction time showed significant main effects of cysteamine (*F*(1, 18) = 22.11, *p* < 0.001) and tunicamycin (*F*(1, 18) = 20.75, *p* < 0.001). Cysteamine pretreatment could attenuate the tunicamycin-induced decreases in interaction with a stranger mouse (*p* < 0.05; Figure 3(c)).

### 3.4. TG2 mRNA and Protein Levels Are Higher in the Middle Frontal Gyrus of ASD Subjects

TG2 mRNA had small but nonsignificant associations with age (*r* = −0.21, *p* = 0.301), pH (*r* = 0.226, *p* = 0.338), and RNA integrity (*r* = 0.296, *p* = 0.206). TG2 protein had small nonsignificant correlations with age (*r* = 0.293, *p* = 0.147) and storage time (*r* = −0.257, *p* = 0.205). These covariates were evaluated in the ANCOVA models. With age, pH, and RNA integrity entered as covariates, the predicted main effect of affection status on TG2 mRNA was significant (*F*(1, 15) = 6.59, *p* = 0.021, Cohen's *d* = 1.10). Overall, the ASD samples demonstrated higher TG2 mRNA levels than the control samples (*p* < 0.05; Figure 4(a)). None of the covariates—age, pH, or RNA integrity—significantly predicted TG2 mRNA in the model.

Age and storage time were entered into the ANCOVA as covariates, but neither covariate significantly predicted TG2 protein levels. The predicted main effect of affection status was significant (*F*(1, 22) = 4.26, *p* = 0.047, Cohen's *d* = 0.89). TG2 protein levels were higher in the middle frontal gyrus of ASD subjects as compared to controls (*p* < 0.05; Figure 4(b)).

Diagnosis of autism is confirmed by the structured ADI-R carried out with the parents. We examined potential associations between TG2 expression and ADI-R scores, which are associated with phenotypic severity in key domains of autism: impairment of social reciprocity, impairment of verbal or nonverbal communication, stereotyped or repetitive behaviors, and abnormality of development.  Table 2 summarizes the correlations of TG2 mRNA and protein levels with ADI-R subscales. The correlations should be considered with caution, given the small size of the ASD sample. Moreover, none of the correlations achieved statistical significance.

## 4. Discussion

We report the first evidence on the role of TG2 in social behavior. We demonstrate that inhibition of TG2 attenuates ER stress-induced deficits in social behavior in mice. We find an increase in TG2 protein levels in the PFC following tunicamycin treatment and demonstrate that overexpression of TG2 in the PFC is sufficient to induce social behavior deficits. In the human brain samples, we find increased levels of TG2 mRNA and protein in the middle frontal gyrus of ASD subjects. Taken together, the findings suggest a novel role of TG2 in modulating social behavior.

Disruptions in PFC neurocircuitry have been implicated in pathophysiology of a number of neuropsychiatric disorders with shared social deficits [33]. For example, hyperconnectivity has been observed among the frontal, temporal, and subcortical regions in gamma frequency ranges in subjects with ASD or schizophrenia [34, 35]. ASD children with greater connectivity exhibited more severe impairment in the social domain [36]. Consistent with this, our recent study revealed hyperconnectivity in the PFC-hippocampus in mice treated with tunicamycin [15]. The effect of TG2 on PFC connectivity remains unknown; however, the attenuation of ER stress-induced social deficits in TG2 shRNA-treated mice suggests that altered TG2 may contribute to PFC neurocircuitry changes.

While a rodent model that fully recapitulates human social behavior is unattainable, the current study validated our previous findings on the role of ER stress in inducing changes in social behavior [15]. ER stress may be one potential mechanism for the synaptic deficits and social behavioral alterations that are observed in several neuropsychiatric disorders including schizophrenia, depression, and ASD [3–7]. Many of the ASD risk factors during pregnancy as well as genetic variations in several synaptic genes implicated in ASD have been shown to induce ER stress [3]. We recently reported increases in the expression of a number of genes involved in the ER stress pathway such as XBP1 and IRE1 in the PFC of ASD subjects [7]. Moreover, increases in ER stress markers have been found in rodent model of depression [4].

The mechanism involved in TG2-mediated social behavior deficits under ER stress is not clear, but TG2 is constitutively expressed in many cell types and is also actively regulated in a cell type-dependent manner. ER stress is known to induce Ca^2+^ release from the ER lumen, and high Ca^2+^ concentrations increase the activity of TG2 [16]. TG2 plays an important role in posttranslational modifications of proteins involved in many neuronal functions [21, 37]. We recently reported that TG2 overexpression in neurons results in a decrease in TrkB levels [22]. Alterations in BDNF signaling through TrkB have been implicated in many neuropsychiatric disorders with shared social deficits [22, 38, 39]. Moreover, BDNF signaling has been shown to rescue neuronal cells against tunicamycin-induced ER stress [40]. It is also plausible that TG2 is involved in other pathways such as histone modification [41], which has recently been shown to regulate social behavior [42]. Thus, multiple posttranslational modifications may be involved in TG2-mediated social behavior deficits under ER stress, which will be investigated in future studies.

The region-specific effects of tunicamycin on TG2 expression are currently unknown. It may be due to region-specific differences in UPR induced by tunicamycin. The lack of effect of tunicamycin on TG2 in the hippocampus suggests that the hippocampus has a more efficient protective mechanism against ER stress-induced increases in TG2 levels. Also, it is important to determine whether tunicamycin induces changes in TG2 at transcriptional level and epigenetic mechanisms contribute to the region-specific effects on TG2 levels. We have recently shown that TG2 is highly expressed in pyramidal neurons in the mouse brain [22]. However, future studies would investigate whether specific cell populations in the PFC are more vulnerable to ER stress.

## 5. Conclusions

We have shown that TG2 in the PFC is a key molecule involved in social behavior deficits under ER stress, findings of interest to better understand the possible involvement of TG2 in the pathogenesis of many neuropsychiatric disorders with social behavior deficits.

## Figures and Tables

**Table 1:** Comparison of autism spectrum disorder (ASD) and control samples on evaluated covariates.

Covariate	ASD	Control	*F*(1, 24)	*p*
M (SD)				
Age (years)	11.80 (5.80)	11.70 (5.71)	0.002	>0.05
PMI (h)	19.00 (10.01)	14.46 (7.83)	1.66	>0.05
Storage time (days)	2828.77 (1434.06)	4286.85 (2302.85)	3.76	>0.05
pH	6.11 (0.26)	5.95 (0.21)	3.00	>0.05
RNA integrity	6.84 (1.92)	5.55 (2.56)	2.13	>0.05

PMI = postmortem interval.

**Table 2:** Correlations between TG2 and autism diagnostic interview subscales.

	ADI-A	ADI-BV	ADI-BNV	ADI-C	ADI-D
TG2 mRNA	−0.319	0.866	0.354	0.371	0.006
TG2 protein	−0.163	−0.002	0.544	−0.625	0.325

ADI-A = social interaction; ADI-BV = verbal communication; ADI-BNV = nonverbal communication; ADI-C = stereotyped behavior; ADI-D = abnormality of development.

## Data Availability

Data supporting the findings are available upon request.
